# Influence of Casein Hydrolysates and Yeast on the Rheological Properties of Wheat Dough

**DOI:** 10.3390/gels8110689

**Published:** 2022-10-26

**Authors:** Ricardo Troncoso Recio, Nelson Pérez Guerra, Ana Torrado Agrasar, Clara Asunción Tovar Rodríguez

**Affiliations:** 1 Department of Analytical and Food Chemistry, University of Vigo, 32004 Ourense, Spain; 2 Department of Applied Physics, University of Vigo, 32004 Ourense, Spain

**Keywords:** bread dough, casein hydrolysates, stress sweep, mechanical spectrum, yield point

## Abstract

The influence of casein hydrolysates (CHs) and yeast on the viscoelasticity of wheat dough at 25 °C were analysed. Three wheat doughs were studied: the unyeasted dough (UYD), the unyeasted dough with CHs (UYD-C) and the yeasted dough (YD). The characteristic parameters in the linear viscoelastic range (LVER) were analysed by stress sweep at 6.3 rad/s: UYD-C dough exhibited higher values of stress (*σ_max_*) and strain (*γ_max_*) amplitudes, and softer gel network (lower complex modulus, G*) comparing with UYD dough. The oscillatory data suggest that CHs would work as (energy and time) stabilising-agents based on the greatest reticular energy (*E* parameter) and the lowest frequency dependence of phase angle (*δ*) at the low frequency range. The rotatory tests show that CHs may act as shear thinning agents in the gluten-starch network, facilitating the solid-fluid transition at the yield point (UYD-C dough). The yeasted dough (YD) exhibited a more shear sensitive structure, evidenced in the highest influence of frequency on the elastic (*G*′) and viscous (*G*″) parameters, and gel to sol transition at 0.23 rad/s was observed.

## 1. Introduction

Developed wheat dough is the material obtained by mixing wheat flour with a proper ratio of water and kneading [1,2]. The energy input provided by the mechanical work during dough making induces critical changes in the protein structure that compose the characteristic gluten fraction of wheat. As a result, a continuous protein network, properly called “gluten” is formed by intermolecular interactions among the gluten proteins mediated mainly by hydrogen bonding, hydrophobic interactions, and covalent (disulfide) bonds [3]. The developed wheat dough may be considered a complex material composed by a homogeneous protein matrix that includes starch crystals as a filler phase.

From a rheological perspective, developed wheat dough is a viscoelastic gel whose parameters are frequency dependent. So, it is a complex system which may be described in a fundamental and quantitative way by studying its viscoelastic properties [4,5]. Moreover, the rheological properties in the linear viscoelastic range (LVER) are related to the structural and compositional characteristics of the material, which in turn provide useful information on the dough quality and product manufacture [6].

Wheat dough is the basis for a number of products, being fermented bread the most important one. In this case, the viscoelastic properties of the gluten network allow retaining the CO_2_ generated during the fermentation of the bread dough by the added yeast. As a result, a characteristic porous, spongy and soft crumb is developed after baking [7].

Different bread products with improved nutritional, functional and organoleptic properties can be obtained by incorporating new ingredients, but they play a relevant technological role in dough processing and development that must be tested [8]. The fortification of bread with proteinaceous ingredients increases the protein content that can balance the wheat amino acid profile, enhancing the nutritional value of the product [8]. Specifically, the incorporation of casein hydrolysates (CHs), which are peptides obtained from the enzymatic hydrolysis of milk caseins, is an interesting strategy to overcome the deficit of wheat proteins in lysine, threonine and methionine [9]. Additionally, CHs are rich in phosphoserine, which is responsible for their mineral binding capacity and their potential to improve calcium bioaccessibility during the gastrointestinal digestion by increasing Ca^+2^ solubility [10,11].

Regarding the technological role of these ingredients, changes in the behaviour of wheat dough upon protein addition have been described. It has been reported [12] that the protein-induced changes in the viscoelastic parameters of wheat dough depend on kind of added protein, because whey proteins increased the viscoelastic moduli (mechanical spectrum) and, in contrast, soy protein decreased the gel strength. Another research [8] observed that the addition of CHs for bread making reduced both the proof time and firmness and increased the volume and softening (10 and 20 min later). Similarly, another investigation [13] showed that CHs in wheat dough produced different rheological characteristics compared to control dough, based on farinograph data. According to the latter researchers, the addition of 5% CHs increased the degree of softening, the dough stability time, the dough development time and the water absorption capacity. So, CHs incorporate a number of ionic compounds due to the NH_4_^+^ and COO^−^ amino acidic groups and amino acids with charged side chains, such as glutamic acid and phosphorylated serines [14]. Additionally, CHs had been successfully used as emulsifiers in the food industry [15]. 

Up to now, the viscoelastic properties and yield point of wheat dough fortified with casein hydrolysates had not been reported. Therefore, the aim of this research was to analyse the structural role of CHs in the wheat dough by studying the rheological magnitudes of the gluten network through fundamental tests. For that purpose, wheat doughs with and without CHs were analysed. A yeasted dough was also explored, to compare both mechanical (pressure of phase gas -CO_2_-) and chemical (CHs) variables that could affect the development of the dough.

## 2. Results and Discussion

To analyse the structural role of the hydrolysates in wheat dough, different fundamental (dynamic and rotatory) tests were applied to three wheat doughs: the unyeasted dough (UYD), the CH-enriched unyeasted dough (UYD-C) and the yeasted dough (YD). The different experiments are described, and their results are discussed below.

### 2.1. Linear Viscoelastic Range (LVER)

The comparison between YD and UYD doughs, indicates the rheological influence of the CO_2_ generated by yeast in the complex (gluten-starch) network. Differences among stress (*σ_max_*) and strain amplitudes (*γ_max_*) were not significant (*p* > 0.05). It can be noted that dough YD exhibited a significantly (*p* < 0.05) higher loss factor (*tanδ*) value than UYD (Table 1), showing a higher viscous component in the fermented dough. That is a normal effect of CO_2_ in the network, whose mechanical pressure partially breaks the gluten network increasing the number of dangling chains or free ends which are dissipative substructures in the network [16], resulting in a less cohesive structure (intermolecular bonds with lower lifetime) in the gluten network [17]. The increase in the viscous modulus produces a subsequent increase in the slope “*a*” (YD vs. UYD) (Table 1). This fit parameter is essentially dependent on both elastic and viscous resistances to deformation [18]. Considering both opposite trends (higher “*a*” and lower *γ_max_* values), no significant differences (*E* parameter) between YD and UYD doughs were found.

On the other hand, CHs in the unfermented wheat dough increased *σ_max_* and *γ_max_* for UYD-C vs. UYD (Table 1). The increase in *γ_max_* is associated with an improvement in the conformational flexibility in the gel network (UYD-C vs. UYD) [17]. The behaviour of these charged peptides in the gluten network (UYD-C) could explain this rheological response. They are characterised by a high degree of hydrolysis (17%) that generates a great number of ions (NH_4_^+^ and COO^−^), and the presence of glutamic acid (charged) and some cysteine residues. So, it is expected that the hydrophilic and ionic amino acids from the hydrolysate increase the electrostatic repulsive forces, producing a subsequent swelling of the viscoelastic network. In these physical conditions, the amount of hydrodynamic water, which would be distributed in several hydration spheres, may be increased [19]. This fact is consistent with the greater viscous component (higher *tanδ*) in UYD-C vs. UYD. The importance of water in the rheological characteristics of dough has also been described by other authors [20,21], who hypothesised that the incorporation of ingredients with high water affinity may reduce gluten hydration and affect the dough’s properties. It has been reported that the absence of enough water during kneading hampers the necessary transformation of the native β-sheet structure of the gluten proteins into β-turns and β-spirals. It has been hypothesised that the reduction of β-turns could generate a more stretched and layered structure of the proteins [22]. So, in the presence of CHs, some depolymerisation of gluten polymers would occur due to molecular distances, rather than interactions of peptides [22]. An enhanced extension would explain the higher strain amplitude (high *γ_max_*) for UYD-C vs. UYD sample which is associated with a greater amount of energy per unit of volume that is needed to reach the limit region of the linear viscoelastic range (higher *E*) [18]. The ordered expansion in the gluten matrix would also include the hydrodynamic and physically entrapped water, which would also explain the increase in the “viscoelastic ductility” of the gluten-starch network, of dough UYD-C vs. UYD.

Additionally, the cysteine residues from the CHs could also hamper the formation of disulfide bridges that occur during dough formation and are directly involved in the dough strength [23]. Here, CHs probably may change the balance between covalent (disulfide) and non-covalent (physical) bonds in gluten reticle. So, CHs reduced the number of disulfide bonds while ionic, ion-dipole interactions increased. As a consequence, a weaker (lower G*) gluten network was formed in UYD-C vs. UYD. 

### 2.2. Mechanical Spectra

**Figure 1:**
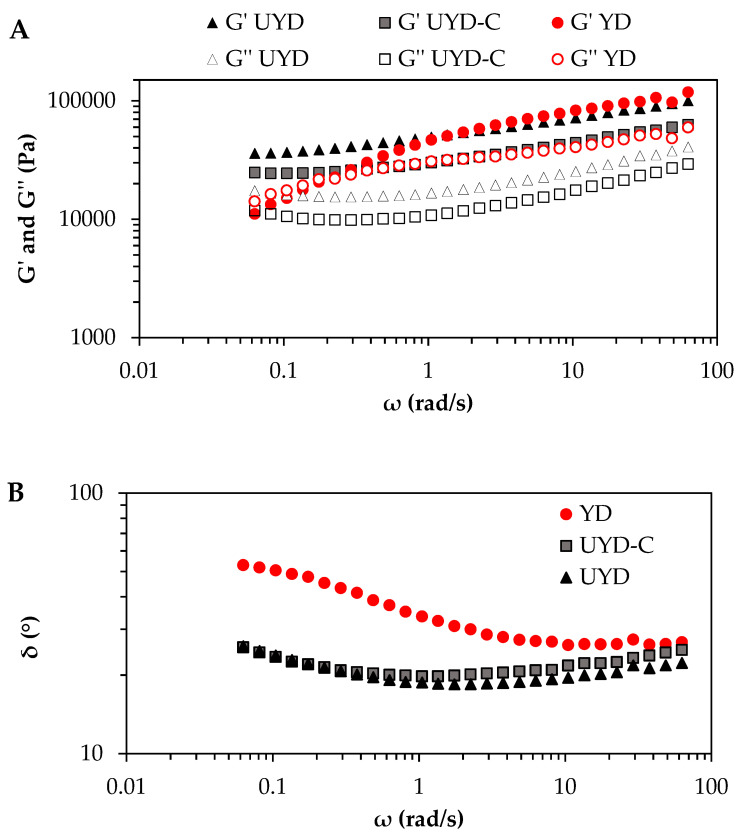
(**A**) Mechanical spectra as a function of the elastic (*G*′) and viscous (*G*″) parameters and (**B**) mechanical spectra as a function of phase angle (*δ*) of unyeasted wheat dough (UYD), yeasted wheat dough (YD) and CH-rich unyeasted wheat dough (UYD-C). T = 25 °C. Empirical data are mean values of 7 replicates.

Mechanical spectra show the influence of frequency on the viscoelastic parameters of doughs. YD sample showed higher frequency dependence than UYD (Figure 1A). Indeed, a gel to sol transition was observed in dough YD at 0.23 rad/s. So, YD network at low frequency range (0.063–0.23 rad/s) exhibited fluid response (sol state) with *G*″ > *G*′, while at higher frequency range (0.49–63 rad/s), a solid-like (gel state) behaviour with *G*′ > *G*″ was observed (Figure 1A). However, dough UYD exhibited gel behaviour (*G*′ > *G*″) for all frequency intervals (Figure 1A). The gel to sol transition at low frequencies (YD), has been induced by combining both mechanical effects (pressure of phase gas -CO_2_- and the sustained shearing at low frequency) expanding and weakening the gluten network.

To complete the analysis of mechanical spectra, the frequency dependence of *δ* vs. *ω* (Figure 1B) was included. Dough UYD showed two trends according to the frequency: at low frequency interval (0.063–0.63 rad/s), *δ* values diminished with increasing *ω*, while at high frequency interval (0.81–63 rad/s), *δ* weakly increased with *ω* (Figure 1B). The values of *δ* for UYD ranged from 18° to 26°, showing a solid-like response in the complete frequencies interval, in contrast with the fluid-like character exhibited by dough YD (*δ* > 45°) at low frequency interval (0.063–0.23 rad/s). These results showed the role of CO_2_ as an additional dispersed phase in the complex (starch-gluten network system). All these data are consistent with those obtained by [4], who showed a consistency decrease in wheat dough by yeast. Similarly, [24] reported lower gel strength and higher *tanδ* for the fermented wheat dough. The latter authors suggested that the mechanical pressure of CO_2_ inflating the dough matrix contributed to the partial breakage of the viscoelastic reticle. They also added that the decreased pH of the dough produced by yeast enhanced the protease activity that can disrupt the gluten network. In agreement with the results of this research, [25] correlated the total gas in the fermented dough with the highest phase angles and they showed lower rigidity in the fermented wheat dough.

CHs decreased both *G*′ and *G*″ for the complete frequency interval (Figure 1A), evidencing a notable decrease in the gel strength in UYD-C vs. UYD, as was also observed in the significantly (*p* < 0.05) lower *G*_0_′ parameter in UYD-C vs. UYD (Table 2). These results converge with the observed softening in the wheat dough fortified with CHs [8,13] and whey protein [12,26]. They suggest again that the presence of cysteine in the CHs weakens the gel network, hampering the formation of disulphide bonds between proteins but maintaining similar frequency dependence (*n*′) (Table 2). So, in this case, the time dependence of the ideal network fraction (*n*′) was similar in both UYD and UYD-C networks (Table 2), indicating that CHs act orderly preserving the initial degree of permanence or temporal stability in the UYD network. The abundance of ionic compounds in CHs could contribute to this behaviour, considering the ion-dipole interactions between CHs and water molecules and the subsequent influence on the water structure, which plays a main role in the forces that stabilise the gluten network [4] including the hypothesis of the ordered extension of the gluten matrix [22].

Regarding *δ* values, two fits of Equation (4) at two frequency intervals were made: at high frequency interval (0.62–63 rad/s) and at low frequency interval (0.063–0.62 rad/s). *δ*_0_ parameters were statistically no distinguishable for UYD-C vs. UYD gels irrespective of the frequency range (Table 2).

However, the *n_p_* exponents exhibited a different trend in both intervals, being *n_p_* > 0 at the high frequency range (Table 2). Thus, *δ* values in doughs UYD-C and UYD increased similarly with increasing *ω*. Conversely, at low frequency range both *n_p_* < 0, being significantly lower (absolute value) in UYD-C vs. UYD (Table 2). Negative values of *n_p_* indicate some increase in the non-ideal network-fraction, that means a higher number of dangling chains or free ends in the gel network [16]. In this case, *n_p_* was significantly less negative for UYD-C vs. UYD indicating greater time stability induced by CHs in the gel network. This fact is in agreement with the higher “viscoelastic ductility” together with conformational flexibility for UYD-C vs. UYD (Table 1). 

### 2.3. Yield Point

**Figure 2:**
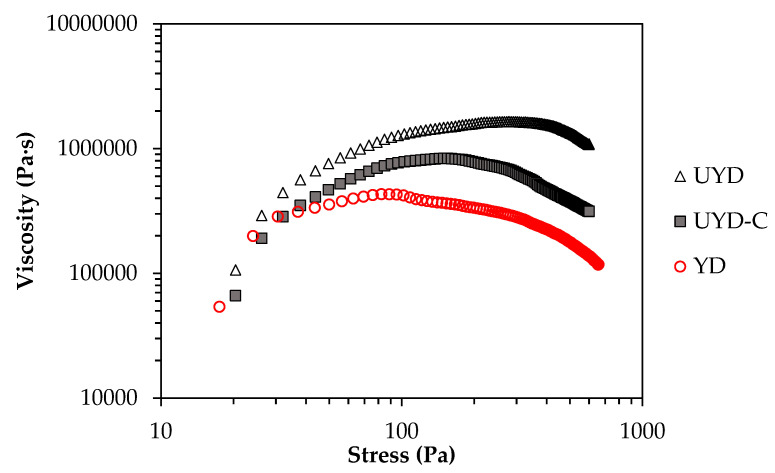
Changes in the apparent viscosity with stress from yield stresses of unyeasted wheat dough (UYD), yeasted wheat dough (YD) and CH-rich unyeasted wheat dough (UYD-C).

Figure 2 shows the comparative apparent viscosities for the three doughs as a function of the applied stress, in which the maximum (critical) viscosity (*μ_c_*) and stress (*σ_c_*) can be noticed. These parameters characterise the rheological change between reversible and irreversible strains based on the potential-energy barrier between inter-particle interactions [27]. Regarding the mechanical effect of CO_2_ pressure, the fermented wheat dough (YD) exhibited lower *μ_c_* and *σ_c_* values than UYD (Table 3) indicating lower consistency, which is equivalent to an easier transition from elastic to plastic deformation in the gluten network. This result reflects the CO_2_ role entrapped in the gluten matrix (dough YD), which expands the gel network [4,24]. The surface tension in the solid–gas interface between the gluten matrix (continuous phase) and CO_2_ (dispersed phase) would produce a brittle boundary surface [28]. This fact would explain the more stress-vulnerable, irregular, and less dense (more porous) structure, as evidenced by the lower critical parameters of yield point in YD vs. UYD. This is in agreement with the network softening in the yeasted dough reported by [4]. The critical strain (*γ_c_*), for dough YD was significantly lower than that of UYD (Table 3). This result is consistent with the more brittle composite of YD vs. UYD, suggesting that the solid-gas interface destabilises the network structure, favouring the irreversible deformation and subsequent intermolecular flow in the network.

Regarding the influence of CHs, dough UYD-C presented a significantly lower *μ_c_* than UYD (Table 3). This result allows to visualise and compare the differences between chemical (CHs) and mechanical (pressure -CO_2_-) softness in the gluten network. The chemical effect is less than that mechanical one. So, although both produce a network softening in gel, CHs kept better the specific properties of the initial wheat dough. The critical stress (*σ_c_*), the critical viscosity *μ_c_*, and critical strain (*γ_c_*) were significantly higher (*p* < 0.05) for dough UYD-C vs. YD (Table 3). This result showed that the structure of sample UYD-C collapsed later than sample YD (Figure 2), which is consistent with the greater temporal stabilisation produced by CHs at low frequencies in mechanical spectra (Table 2).

## 3. Conclusions

The fortification of wheat dough with casein hydrolysates (CHs) expanded the gel network orderly, resulting in a more energy stable structure, as was evidenced in the increase of conformational flexibility (higher *γ_max_*) and the “viscoelastic ductility” (higher *E*) of the CH-rich network. 

The comparison between both mechanical (CO_2_ pressure) and chemical (CHs) agents in the unfermented wheat doughs indicated that the dispersed gas phase (CO_2_) disrupted the net structure, resulting in a more open, more transient (frequency dependent), and less cohesive gluten network that experienced gel to sol transition at low frequencies. However, the chemical agents (CHs) softened the network, producing a notable temporal stabilisation at low frequencies, showing the own structural-benefit in the wheat dough. The higher yield parameters (*σ_c_*, *μ_c_*, *γ_c_*) in dough UYD-C vs. YD evidenced a greater viscoelastic character in a more cohesive-entangled structure when CHs were introduced in the wheat dough. 

Summarising it may be concluded that high values of strain amplitude (*γ_max_*), reticular energy (*E*), critical stress (*σ_c_*) and critical viscosity (*μ_c_*) indicate a flexible, energy stable, and consistent dough. Likewise, low power law exponents n′ and n_p_ are indicative of a time stable and consolidated structure of dough. 

The results of the present work suggest that the addition of CHs (4.3 wt%) to wheat dough causes changes in its rheology by different mechanisms that prevent and reinforce the gluten network simultaneously. So, the development of wheat dough-based foods fortified with casein hydrolysates must consider and evaluate the effect of this ingredient on the textural characteristics of the final product according to dose and method of elaboration. 

## 4. Materials and Methods

### 4.1. Fortification Ingredients

Casein hydrolysates (CHs) under the commercial name Hyvital Casein Phosphopeptides were acquired from Friesland Campina Domo (Amersfoort, The Netherlands). The product contains casein hydrolysates at a 17% degree of hydrolysis, presenting 600 Da as the average molecular weight, being 24% casein phosphopeptides. The composition of the product is 91.3% protein, 6.2% ash and moisture below 5.0%. Among other amino acids, the product contains 209 mg/g glutamic acid and 3 mg/g cysteine [29].

The following ingredients were used to make the wheat dough samples: wheat flour (Haribericas XXI S.L., Andalucía, Spain) which contains 10.3% protein, 13.6% water and 0.59% ash; fresh yeast (Lesaffre Ibérica S.A., Valladolid, Spain) which contains 33% solid material, of which, 43% is protein; tap water, and refined dry salt (Mercadona, Valencia, Spain).

### 4.2. Samples

Three wheat doughs were studied: unyeasted control dough (UYD), yeasted dough (YD), and the CH-rich unyeasted dough (UYD-C). The concentration of casein hydrolysates was established in 4.3 wt%. This specific value of CHs concentration has been selected after numerous previous proofs to achieve a particular rheological state: more fluid and stickier for wheat-starch doughs [30], which is specifically relevant for studying in a model of gluten network, such as wheat dough. The formulations and composition of the three doughs are given in Table 4.

### 4.3. Dough Sample Preparation

The wheat dough samples were prepared by mixing for 2 min wheat flour, water and salt in a planetary kneader (Sammic BM-5, Azcoitia, Spain) at low speed (130 rpm). The speed was then increased up to 420 rpm for 3 min. Yeast was added (when applicable) and all the ingredients were mixed for 5 additional min without changing the speed (420 rpm). The wheat dough obtained was slapped and then allowed to ferment for 1 h at room temperature and stored in a fridge at 4 °C. The measurements were carried out during the following 5 days, keeping the chilled samples packed at 4 °C during the intervening period.

### 4.4. Rheological Tests

Small amplitude oscillatory shears (SAOS) were done by a RS600 Haake-rheometer (Thermo Electron, Karlsruhe, Germany). The measurements were obtained using a steel plate-plate PP20 (20 mm diameter and 1 mm gap). Samples were disks of 20 mm diameter and thick 1 mm. They were rested for 15 min before analysis to achieve thermal and mechanical stabilisation at the initial time of the rheological measurements. Steel solvent trap was used to avoid evaporation during testing. Temperature was measured by a Peltier system in the lower plate (25.0 ± 0.1 °C) for all rheological tests.

#### 4.4.1. Linear Viscoelastic Test

SAOS experiments were carried out at increased stresses, at constant frequency (6.28 rad/s). To obtain the linear viscoelastic range (LVER), the stress sweeps were programmed by increasing stress (*σ*) from 25 Pa to 1500 Pa. 250 continuous points were recorded. Values of the storage parameter (*G*′), viscous parameter (*G*″) and loss factors (*tanδ* = *G*″/*G*′) were measured. The limit values of the LVER have been obtained through the complex modulus (G*) with a deviation interval ± 10% [31].

The structural stability of the dough was analysed on the basis of the linear relationship between stress (*σ*) vs. strain (*γ*) in the LVER i.e., according to Equation (1).

(1) σ=a·γ+b

The parameter “*a* (Pa)” provides the net resistance to the (elastic and viscous) deformation in the LVER. The constant “*b*” (Pa) is the initial shear stress at *γ* = 0. Equation (1) might be integrated according to Equation (2).

(2) $$E=\int_{0}^{\gamma_{\max}}\left(a\cdot \gamma +b\right)\cdot d\gamma$$

The “*E*” value (J/m^3^) gives the area under the linear relationship between *σ* and *γ* from *γ* = 0 to *γ = γ_max_*. This area provides the total energy (per volume unit), which is needed to reach the limit of the reticular deformation in the linear viscoelastic zone [18]. Thus, the *E* parameter may be considered as a measurement of the “*viscoelastic ductility*” of the gel network in the LVER. This is an analogous mechanical-effect of resilience for a compression test [32].

#### 4.4.2. Mechanical Spectra

Frequency sweep gives the time stability of samples in terms of the frequency dependence of *G*′ and *G*″. A harmonic stress at small strain (*γ* = 0.1%) in the LVER was applied to gels. The viscoelastic moduli were determined between 0.0628 and 62.83 rad/s at 25 °C.

Mechanical spectra of samples were shown through the frequency dependence of elastic modulus (*G*′) and the phase angle (*δ*). Both parameters were fitted to the angular frequency (*ω*) by the power-law fit (Equations (3) and (4)).

*G*′ = *G*_0_′ *ω*^*n*′^ (3)

(4) $$\delta =\delta_{0}\cdot \omega^{n_{p}}$$

where *G*_0_′ and *δ*_0_ are the elastic modulus, and phase angle at 1 rad/s respectively. These are the basic parameters, which describe the structural domain in the gel network. *n*′ and *n_p_* describe the temporal domain, showing the influence of the angular frequency on the *G*′ modulus and *δ*, respectively [17].

#### 4.4.3. Yield Point

The yield stress is a rotatory test, which was carried out from 20 to 600 Pa (100 steps in a continuous mode) during 10 min at 25 °C. This test provides two characteristic parameters: the critical viscosity (*μ_c_*) and critical stress (*σ_c_*), which determine the point at which a structural collapse in the gel network occurs. So, after the yield point, the material has been irreversibly deformed and it may flow under small change of the applied stress [27]. 

### 4.5. Statistical Analysis

Viscoelastic data are shown as mean values of seven replicates, and they were statistically analysed by the expanded uncertainty limits (EUL). It is the highest and lowest deviation from the mean. Data were considered significant when mean values differed at *p* < 0.05 (Student’s *t*-test).

## Figures and Tables

**Table 1:** Limit parameters of the linear viscoelastic region at 25 °C: limit stress (*σ_max_*), limit strain (*γ_max_*), complex modulus (G*), loss tangent (*tanδ*), as well as the fit parameters of Equation. (1) (*a* and *b*) and result of the integral (*E*, from Equation (2)) of unyeasted wheat dough (UYD), CH-enriched unyeasted wheat dough (UYD-C) and yeasted wheat dough (YD). T = 25 °C.

Sample	*σ_max_* (Pa)	*γ_max_* (%)	G* (kPa)	*tanδ*	*a* (kPa)	*b* (Pa)	r^2^	*E* (J·m^−3^)
UYD	148 ± 15 ^a^	0.153 ± 0.021 ^a^	98 ± 13 ^ab^	0.416 ± 0.015 ^a^	95.00 ± 0.41 ^b^	5.34 ± 0.31 ^a^	0.998	0.116 ± 0.016 ^a^
UYD-C	189 ± 19 ^b^	0.315 ± 0.057 ^b^	72 ± 20 ^a^	0.573 ± 0.012 ^b^	59.67 ± 0.30 ^a^	8.44 ± 0.44 ^b^	0.997	0.316 ± 0.057 ^b^
YD	148 ± 15 ^a^	0.122 ± 0.029 ^a^	143 ± 36 ^b^	0.536 ± 0.028 ^b^	120.31 ± 0.91 ^c^	9.20 ± 0.52 ^b^	0.994	0.098 ± 0.023 ^a^

Data are mean values ± expanded uncertainty limit (EUL). ^a^–^c^: different superscripts within columns show significant differences (*p* < 0.05) among samples.

**Table 2:** Elastic parameter at 1 rad/s (*G*_0_′), *n*′ exponent (Equation (3)) and phase angle (*δ*) (Equation (4)) at high and low frequency intervals of unyeasted wheat dough (UYD) and CH-rich unyeasted wheat dough (UYD-C).

Sample	*G*_0_′ (kPa)	*n*′	*δ*_0_ High Range	*n_p_* High Range	*δ*_0_ Low Range	*n_p_* Low Range
UYD	50.5 ± 6.0 ^b^	0.153 ± 0.003 ^a^	18.03 ± 0.67 ^a^	0.043 ± 0.005 ^a^	17.74 ± 0.79 ^a^	−0.130 ± 0.004 ^b^
UYD-C	31.7 ± 2.8 ^a^	0.145 ± 0.005 ^a^	19.32 ± 0.83 ^a^	0.053 ± 0.004 ^b^	18.6 ± 1.4 ^a^	−0.105 ± 0.008 ^a^

Data are mean values ± expanded uncertainty limit (EUL). ^a^,^b^: different superscripts within columns show significant differences (*p* < 0.05) between samples for each parameter.

**Table 3:** Values of critical viscosity (*μ_c_*), critical stress (*σ_c_*) and critical strain (*γ_c_*) of yeasted wheat dough (YD), unyeasted control wheat dough (UYD) and CH-rich unyeasted wheat dough (UYD-C).

Sample	*μ_c_* (kPas)	*σ_c_* (Pa)	*γ_c_* (%)
YD	437 ± 206 ^a^	83 ± 12 ^a^	1.34 ± 0.24 ^a^
UYD	1717 ± 652 ^c^	280 ± 58 ^c^	3.31 ± 0.51 ^c^
UYD-C	848 ± 90 ^b^	166 ± 17 ^b^	2.47 ± 0.20 ^b^

Data are mean values ± expanded uncertainty limit (EUL). ^a^–^c^: different superscripts within columns show significant differences (*p* < 0.05) among samples.

**Table 4:** Formulations used for the wheat doughs: yeasted wheat dough (YD), unyeasted control dough (UYD) and CH-rich unyeasted dough (UYD-C).

Ingredient (g/100 g)	YD	UYD	UYD-C
Wheat Flour	70 (100%)	65 (100%)	62 (100%)
Water	27 (39%)	34 (52%)	32 (52%)
Salt	1.3 (1.9%)	1.2 (1.9%)	1.2 (1.9%)
Yeast	1.4 (2.0%)	-	-
CHs	-	-	4.3 (6.9%)

Percentages are referred to the flour content.

## Data Availability

The data presented in this study are contained within the article.
